# Treatment of task-specific dystonia in sports: A systematic review

**DOI:** 10.1016/j.prdoa.2024.100245

**Published:** 2024-02-28

**Authors:** B. Nijenhuis, E. van Wensen, M. Smit, T. van Zutphen, J. Zwerver, M.A.J. Tijssen

**Affiliations:** a Department of Neurology, University of Groningen, University Medical Centre Groningen, Groningen, The Netherlands; b Expertise Center Movement Disorders Groningen, University of Groningen, University Medical Centre Groningen, Groningen, The Netherlands; c Department of Neurology, Gelre Ziekenhuizen Apeldoorn and Sports Dystonia Centre, Apeldoorn, The Netherlands; d Sports Valley, Sports Medicine, Gelderse Vallei Hospital, Ede, The Netherlands; e University of Groningen/Faculty Campus Fryslân, Leeuwarden, The Netherlands; f Center for Human Movement Sciences, University of Groningen, University Medical Center Groningen, Groningen, The Netherlands

**Keywords:** Task Specific Dystonia, Sports, Treatment, Intervention

## Abstract

**Introduction:**

Task specific dystonia is a movement disorder only affecting a highly practiced skill and is found in a broad set of expert movements including in sports. Despite affecting many sports, there is no comprehensive review of treatment options, which is in contrast to better studied forms of task specific dystonia in musicians and writers. For this reason, studies involving an intervention to treat task specific dystonia in sports were systematically reviewed, with special attention for the quality of outcome measures.

**Methods:**

The PICO systematic search strategy was employed on task-specific dystonia, and all synonyms. Inclusion criteria were peer reviewed published studies pertaining to sports, studies with a measurement and/or intervention in TSD, all in English. We excluded abstracts, expert opinions, narrative review articles, unpublished studies, dissertations and studies exclusively relating to choking. We included case reports, case studies and case-control studies.

**Results:**

In April 2022 Pubmed, Embase, Web of Science, and Psychinfo were searched. Of the 7000 articles identified, 31 were included that described psychological and invasive and/or pharmacological interventions. There was a lack of formal standardized outcome measures in studies resulting in low quality evidence for the effectiveness of treatment options. A descriptive synthesis showed emotional regulation was effective, but was exclusively tried in golfers. Interventions like botulinum toxin or pharmacology had a similar effectiveness compared to studies in musicians dystonia, however there was almost no formal evidence for these treatments.

**Conclusion:**

The quality of studies was low with a lack of standardized outcome measures. Future studies with larger cohorts and quantitative outcome measures are needed to improve understanding of treatments for task specific dystonia in athletes.

## Highlights

• We systematically reviewed treatments for task-specific dystonia in sports.
• This is the first comprehensive review of treatments for task-specific dystonia in sports.
• Invasive/non-invasive treatment studies require more standardized outcome measures.
• More diverse treatment strategies should be trialed in a greater diversity of sports.

## 1 Introduction

Dystonia is defined as a “collection of movement disorders characterized by sustained or intermittent muscle contraction causing abnormal, often repetitive, movement, postures, or both”[1]. In certain cases, dystonia is limited to a specific highly practiced skill. Known as task-specific dystonia (TSD), its exact pathophysiology is still unclear, though evidence it arise due to changes in the sensory-motor system caused by over-practicing, equipment change, or injury[2]. These changes are thought to negatively impact motor-control networks specifically formed for the practiced skill, and are therefore partly etiologically distinct from other forms of dystonia [3], [4]. Additionally, evidence has shown maladaptive dis-inhibition of the basal ganglia and motor cortex is also involved and may result in overactivity intra-hemispherically[3]. This flawed motor-control circuitry and overactivity is thought to result in muscular overflow in peripheral non-active muscles during complex motor tasks. The result is the most common symptom of TSD: excessive maladaptive muscle contractions with subsequent abnormal postures exclusive to highly skilled and repetitive movements [2].

TSD affects a broad set of motor-related pursuits as performed by typists, painters, hairdressers, and watchmakers [4], [5]. In skills with higher motor-cognitive demands, such as professional musicianship it is even more common[6]. For this reason, it also presents in many sports, as they offer another very competitive and complex test of motor-control. TSD has been observed in running, pistol shooting, golf, and table tennis [7]. More recently new forms have been identified including manifestations in billiards, darts, rowing, cricket and speed skating. Some sport-related forms of TSD seem to be quite prevalent, sometimes with a sport specific terminology, for example studies in golf report a prevalence of Yips (Type 1, golf specific TSD) ranging from up 22 % to almost 50 % amongst avid players [8], [9], [10] (results are survey-based, cursory and need more concrete epidemiological evidence). Despite the high prevalence, a recent review concluded that the study of TSD in sports is still in its infancy[11], with far fewer research studies conducted compared with musician’s cramp, which has a prevalence of 1–12 %, and writer’s cramp with a prevalence of 2.7 out of 1 000 000 [12], [13].

As research studies are generally scarce, the optimal treatment strategy for TSD in sports has also not been elucidated, with recommendations by clinicians often being based on anecdotal evidence[14]. There are few robust studies on treatments for TSD in sports, with most reviews being narrative in nature [15], [16], [17]. Therefore, the purpose of this study was to systematically review all original research describing interventions in the management of TSD in sports, with attention for the outcome measures used. It is our aim that this review contributes to a better understanding of the heretofore under-investigated treatment protocols for TSD in sports, and raises awareness regarding the necessity for further research.

## 2 Methods

### 2.1 Search strategy

The PICO [18] search strategy was used employing the following search criteria: **P**opulation: subjects with TSD in sports; **I**ntervention/Indicator: interventions to rehabilitate TSD in sports; **C**omparison/Control: none/any comparators; and **O**utcome: Pre and post-intervention measures of rehabilitation in subjects with TSD in sports. A systematic search strategy was employed until 06/06/2022 of TSD, and all synonyms for task-specific dystonia, in sports (i.e. yips or occupational cramp) (See Appendix A for search strategy). This search in Pubmed was also translated into Embase, Web of Science, and Psychinfo (See Appendix A). Additionally, for each retrieved article, all citations were reviewed and eligible articles were retrieved.

Studies were included if they met all the following criteria: 1) Peer reviewed published studies pertaining to sports; 2) Studies with a measurement of TSD; defined as an abnormal motor activity occurring during the execution of a skilled sport-specific task. Certain studies mainly in baseball and golf refer to TSD in sports as ‘type 1 yips’, placing it as one subtype within a larger psycho-neuromuscular disorder (the yips) that sometimes includes dystonia (type 1 yips), and sometimes does not (type 2 yips). Articles labeled type 1 yips were considered to be TSD and included. 3) Studies with an intervention focusing on rehabilitation; defined as any physiological (neurological or peripheral) or psychological intervention employed to improve symptoms of TSD in sports 4) Articles in English with no restriction on publication date. We included case reports, case studies and case-control studies. We excluded articles if they were: 1) Abstracts sans full texts, expert opinions, narrative review articles, unpublished studies and dissertations; 2) Studies exclusively relating to choking. All papers were independently screened by two authors (BN and EW) by title, abstract and by keywords using Rayyan Qatar Computing Research Institute platform [19]. The level of agreement was measured with Cohen’s Kappa. The full text version of articles that met the inclusion criteria were acquired and assessed. Disagreements were resolved through consensus in consultation (MT and MS). If further information was required, authors were contacted for clarification.

### 2.2 Data extraction

Data was extracted from eligible articles, including participant demographics, study design, follow up period, type of intervention, type of outcome measures (standardized or subjective), statistical analysis, results and conclusion. Studies were categorized into two forms of intervention, 1) Paramedical; comprising psychological (emotional regulation or motor retraining), or physical-therapy based interventions. 2) Medical; comprising botulinum toxin A (BTX-A), (oral) pharmacology or surgery. Summary statistics were aggregated from extracted data such as the frequency of different study designs, and the prevalence of standardized outcome measures.

### 2.3 Quality assessment

Due to the lack of cohort studies and randomized controlled trials (RCTs), we assessed the methodological quality of included studies with the McMaster Critical Review Form for Quantitative Studies (MCRF) [20]. Based on the MCRF questions, summary scores were calculated to rate the quality of each study using a consistent comparison across different research designs [14]. In agreement with previous studies, a percentage score of 50 % or less was perceived as a methodologically low score, while between 51 % and 75 % was considered moderate and greater than 75 % as high (See Appendix B for details). A meta-analysis and or subgroup analyses was not conducted due to the absence of homogeneous studies with sufficiently large sample sizes and standardized outcome measures.

The PRISMA 2020 (Preferred Reporting Items for Systematic Reviews and Meta-Analysis) [21] guidelines were followed strictly in structuring this systematic review, and the protocol was registered in PROSPERO (Registration number: CRD42021261631).

## 3 Results

**Fig. 1:**
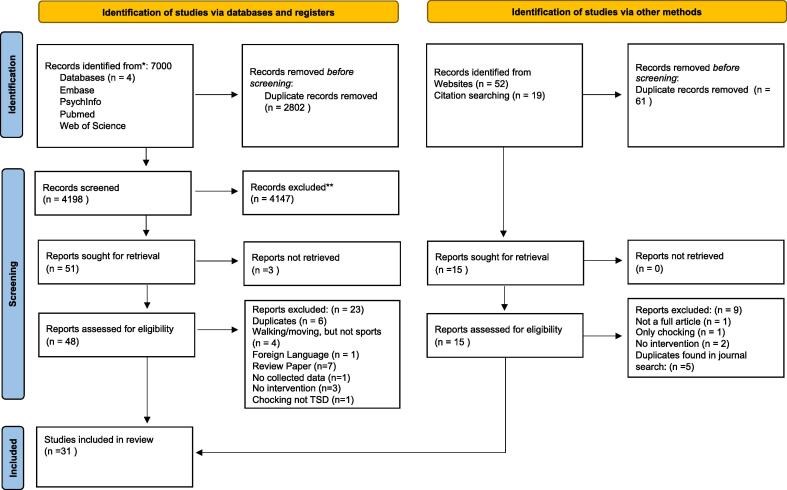
Search protocol for filtering studies.

From all sources, 31 articles were retained for this review (complete search protocol in Fig. 1). 7000 records were identified in our systematic search, of which 2802 were duplicates. Fifty-one articles met the criteria, of which three were not retrieved due to being unavailable, seven were review papers, six duplicates, four not directly sports-related, one was in a foreign language, one had no outcome variable, three had no intervention and one was not a TSD, leaving 25 included. Agreement between the two examiners following initial screening was a Cohen’s Kappa score of 0.64, with a 99.36 % agreement, signifying substantial agreement. Additionally, 71 articles were identified from citation screening, of which 61 were duplicates and, after final assessment via group discussion, 6 remained, resulting in a total of 31 articles.

### 3.1 Study characteristics

In total, 74 participants were involved in interventions to rehabilitate their sport-specific TSD. The majority of studies were single-participant, with 14 case reports and 7 single case studies. Study populations ranged from 1 to 14 with a median of 1 participant per study. Golf was the sport with the most studies on sport-specific TSD with 12 studies [17], [22], [23], [24], [25], [26], [27], [28], [29], [30], [31], [32], second was running with 5 studies [33], [34], [35], [36], [37], and following these a series of diverse sports with no more than three studies per sport including Table Tennis [38], [39], Baseball [40], [41], Billiards[42], [43], Juggling [29], [39], [44], Rowing[45], [46], Tennis [39], [47], Pistol Shooting [48], Cycling[49], Distance Walking [15], and Flamenco Dancing [50]. Table 1 summarizes all the articles on TSD in Sports (See Appendix C and D for more details).

**Table 1:** 

**TSD-S**	**N**	**Is**	**Ps**	**Intervention**	**Effect**	**MCRF**
			♂/♀			**(out of 16)**
**Non-Invasive**						
Golf	9	7	27 ♂	Looking at the hole	+	13
				SFGI	+/+/+	7/9/9
				EMDR	+	11
				EFT	+	6
				Acupuncture	+	10
				PPR	+	7
				SMR	+	7
Running	3	4	15 (9♂/6♀)	Physical Therapy	+	10
				Weighted Pack	+	10
				SMR	+	11
				Sensory Trick	+	7
Rowing	1	1	1♂	CBT	+	8
**Total**	13	12	43			

**Invasive/Pharm.**						
Golf	3	7	20♂	BTX-A (2)	+/–	8/7
				DBS	++	7
				PPL	+	13
				THP/CLZ/BCF/TZD	–/–/–/–	7
Running	6	9	26 (12♂/14♀)	BTX-A (5)	4 +/ 1 –	10/5/13/7/7
				VoTM	CR	7
				CLZ (3)	+/+/–	10/13/7
				L-Dopa (4)	+/ 3–	10/5/7/7
				CBZ (2)	+/–	10/7
				THP (4)	2 +/ 2 –	10/7/7/7
				DZP	+	7
				BCF	–	7
Rowing	1	1	1♂	L-Dopa	–	3
Table Tennis	2	3	2 (1♂/1♀)	VoTM (2)	CR/+	7/10
				THP/CLZ	–/–	7/7
Dancing	1	2	1 ♀	L-Dopa/ BZD	–/–	3
Tennis	2	2	3 ♂	VoTM	+	10
				THP	+	4
Juggling	3	3	3♂	BTX-A	+	3
				VoTM	+	10
				MMT	+	4
Baseball	2	4	3♂	BTX-A	–	6
				LDCi	+	6
				THP/TBZ	–/–	3/3
Billiards	2	4	2♂	BTX-A (2)	+/–	5/4
				PPL/BZD/ACs	–/–/–	4/4
Pistol shooting	1	1	1♂	BTX-A	–	4
Total*	21	17	62 (46 ♂/16♀)			

N = Number of articles, Is = Number of interventions, Ps = Number of participants, MCRF = McMaster Critical Review Form, SFGI = Solution Focused Guided-Imagery, EMDR = Eye Movement Desensitization Reprocessing, EFT = Emotional Freedom Technique, PPR = Pre-Performance Routine, SMR = Sensory Motor Retraining, CBT = Cognitive Behavioral Therapy, BoNT/A = Botulin-Toxin type A injections, DBS = Deep Brain Stimulation, PPL = Propranolol, THP = Trihexyphenidyl, CLZ = Clonazepam, BCF = Baclofen, TZD = Tizanidine, VoTM = Ventro-oral Thalamotomy, CBZ = Carbamazepine, DZP = Diazepam, BZP = Benzodiazepine, MMT = Memenatine, LDCi = Lidocaine-injections, TBZ = Tetrabenazine, ACs = Anticholinergics, – = no effect, + = some effect, ++ good effect, CR = Complete Remission, * = doubles included.

### 3.2 Study quality

The general quality of studies in TSD in sports was low, with a combined mean MCRF score of 49 % (STD 20 %). The non-invasive interventions with an MCRF of 59 % were more methodologically robust compared to invasive and or pharmacological interventions with an MCRF of only 42 %. Looking at individual characteristics of MCRF scores revealed many general deficiencies. All studies lacked any assessor blinding or power calculation to justify sample sizes. Three out of 31 studies presented results in terms of statistical significance [23], [28], [35]. Only 12 of 31 studies documented ethical approval or informed consent[17], [23], [24], [25], [26], [32], [33], [35], [37], [38], [39], [42]. There were no properly matched control groups. Interventions were often not described in detail, hampering future replication. No studies reported power calculations.

### 3.3 Outcome measures

#### 3.3.1 Paramedical

Eight paramedical studies used standardized outcome measures. Performance in golf putting was used in four studies [24], [25], [26], [30], three used kinematics [23], [27], [31], and one used EMG [23]. A psychological survey (the Bangor Sports Psychological Skills Inventory) and a checklist (the Pre-Performance Routine Checklist) were used in one study [28]. The remaining paramedical studies used subjective, non-standardized outcome measures. [22], [33], [36], [46].

#### 3.3.2 Medical

Standardized outcome measures were used in three invasive and/or pharmacological interventions. EMG and Kinematics were used in two studies [23], [35], performance measures in one study [23]. Two studies used surveys, the The Lower Extremity Functional Scale (LEFS) [35] and the Task-Specific Focal Dystonia Scale (TFDS) [39]. Non standardized outcome measures were used in the remainder of invasive and or pharmacological studies[15], [17], [29], [32], [33], [34], [37], [38], [40], [41], [42], [43], [45], [47], [48], [49], [51], [52].

### 3.4 Interventions and effectiveness

#### 3.4.1 Paramedical

Seven paramedical studies testingemotional regulation techniques showed possible efficacy in reducing symptoms of TSD in athletes. This included cognitive behavioral therapy(CBT) [46], eye movement desensitization reprocessing (EMDR) [26], emotional freedom technique (EFT) [31], pre-performance routine (PPR) [28] and three instances of solution-focused guided imagery (SFGI) [24], [25], [26], [28], [30], [31], [46]. Sensory motor retraining was trialed in two studies, but the protocol was abandoned in one [35] and showed immediate positive effects in the other [27]. Acupuncture[22], physiotherapy[33], and sensory trick[36], [53] showed possible efficacy.

#### 3.4.2 Medical

BoNT/A showed improvement in seven out of 12 studies [17], [33], [34], [35], [37], [43], [44]. Oral pharmacology seemed effective in six out of 14 studies[23], [29], [33], [35], [37], [47]. Ventro-oral thalamotomy and deep brain stimulation (DBS) seemed highly effective with four studies reporting improvements in patients in a wide range of sports. In three studies a total of six patients (three tennis players, a table tennis player and a juggler and a runner) reported significant improvement after ventro-oral thalamotomy[38], [39], [49]. DBS was successful in the case of a golfer [32].

## 4 Discussion

### 4.1 Main findings

The aim of this study was to systematically review all original research describing interventions in the management of TSD in sports. Thirty-one studies involving ten different sports were found treating a total of only 74 affected participants. Twenty-one studies were medical: invasive and/or pharmacological and 13 were paramedical. Despite the wide range of sports involved and differing intervention strategies, average quality of studies was low (an MCRF score of under 50 %) making any quantitative assessment of treatment effectiveness difficult. The principal deficiency was a lack of quantitative outcome measures and statistics to test the efficacy of treatments, and instead relying on anecdotal evidence. An additional complication was the heterogeneity of interventions, where a large number of different interventions have been trialed, but few have been replicated across multiple forms of TSD. In these low quality studies, the effectiveness of interventions was generally in line with other form of TSD, showing generally positive results for paramedical interventions and mixed results for medical studies. Taken together, our results showed there is no conclusive evidence for an effective treatment strategy for TSD in sports, and more standardized measures of treatment outcomes are needed.

### 4.2 Study quality

TSD in sports is a troublesome condition for skilled athletes that has been insufficiently studied using standard outcome measures and statistics compared to other forms of TSD. This is partly due to the difficulty in finding subjects in rarer forms of TSD. However as seen in studies in other forms of TSD that may be equally rare (musician’s dystonia) [12], [13], it is possible to collect more robust cohorts and use objective measures and statistics more often [54], [55], [56]. For instance a review found eight instances of SMR trialed in musicians[54], whereas we found only two in sports where only one completed the protocol. Another recent systematic review found 75 studies of botulinum toxin injection in writers and musicians, of which three were high quality double blind RCTs [57], [58], [59] and nine open label trials [55]. Comparatively in the 12 sports-related studies we found, there were zero RCTs and only one open-label design [23]. This re-emphasizes the low quality of intervention studies in TSD in sports in comparison. This over reliance on subjective and anecdotal evidence thus results in low reliability and generalizability, and highlights the need for more and better research studies.

### 4.3 Effectiveness

The reported effectiveness of emotional regulation studies was high with all reviewed interventions improving symptoms of TSD in seven studies. It is important to note that these studies are exclusively on golfers. Reviews have noted choking is a common performance deficit in golf, and sometimes interacts with TSD, making it unclear whether these emotional regulation techniques would work equally well in other sport-related TSD [11]. Other paramedical interventions seemed also successful, but these studies were too few to draw broader conclusions. It is notable that SMR was only trialed twice in TSD in sports[27], [35], indicating it is under-represented considering the technique has been especially effective in musician’s dystonia [54].

The effectiveness of invasive and/or pharmacological studies in athletes were similar to studies in musicians and lower than in writers. Seven out of 12 (58 %) of studies of botulinum toxin in sports were effective, which was less than the 73 % efficacy shown in RCTs of 139 patients with occupational, writer’s and musician’s dystonia [60]. This lower effectiveness in sports may be due to the intricate movement demands and strength requirements compared to other forms of TSD. A similar reduced efficacy (54 %) [61] has been shown in musicians specifically, presumably for the same reason [61], [62], [63]. Pharmacological interventions were effective in 6/14 [23], [29], [33], [35], [37], [47] studies. Unlike BTX-A, evidence for pharmacology in other forms of TSD is scarcer with no systematic reviews. Narrative reviews mention inconsistent results frequently accompanied by intolerable side effects [4], [61], [64], which may partly explain the low effectiveness in sports. Surgery was highly effective in sports, reporting unanimous positive results for both ventro-oral thalamotomy and DBS. This agrees with results of successful surgeries in other forms of TSD, particularly in musicians [49]. Although highly effective, these treatments carry significant risk of complications like temporary or permanent dysarthria and other motor deficits [5], [65], [66]. Despite this, recent reviews have suggested favoring surgery in highly coordinated sports, arguing that botulinum toxin may cause excessive losses in coordination making its use untenable for expert performers [55].

### 4.4 Implications for future research

The majority of studies in our review were single cases, indicating a need for prospective studies in larger cohorts. This is challenging as TSD is rare making RCT designs often unfeasible. An alternative is crossover RCTs, where groups are exposed to different treatments regiments sequentially. In instances where only single-case designs are feasible, we recommend using new forms of Bayesian analysis that allow for statistical inferences to be made, even n = 1 style designs[67]. Finally in all studies, interventions should not be trialed simultaneously [33], [42].

Irrespective of study design, researchers should consider using standardized outcome measures on a sport by sport basis due to the diverse physical demands that are specific to different sports. For example, in a case of TSD in baseball botulinum toxin lead to performance deficiencies [41] (likely due to the high strength requirements of the sport), while in a case of billiards it was very effective[43]. To investigate inter-sport differences in effectiveness, outcome measures could include biomechanics (EMG, kinematics), performance (sport-specific metrics such as successful putts in golf, shots on target in darts), and surveys (partly using standardized improvement scales with addended sections that are sport-specific). Therefore we also recommend the use of protocols to reduce heterogeneity in study design and interventions, so that researchers will be more able to measure their efficacy in a standardized manner. This would be a key improvement over clinician’s current reliance on treatment protocols based on studies of other sports, writers and musicians.

Interventions for different forms of TSD should not cluster around particular skills, such as sensory motor retraining being rare in sports, whereas it has been successful in musicians and writers [2], [54]. Conversely, SFGI has been effective in golfers [24], [25], [26], but not adopted to treat other sports or TSD more broadly. These gaps in the research offer a promising opportunity for future treatments. For example, based on aforementioned results, a sequential multiple assignment randomized trial in a large group of golfers, with botulinum toxin and sensory motor training to measure there efficacy separately.

### 4.5 Limitations

It is important to recognize limitations to current research in TSD interventions in sports, specifically in the yips. The yips is a movement disorder where there are two distinct forms of pathophysiology, 1) psychological, where fear causes a loss of ability and 2) physiological, where multiple factors lead to dystonic symptoms. Although there is also significant overlap as remarked by Clarke et al. [11], it remains a challenge to differentiate them diagnostically at the current state of art. We attempted to exclude psychological yips by excluding all chocking articles where dystonia was not mentioned, however the authors concede this still leaves the possibility for confusion as to whether psychology was a factor among many in developing dystonia (as is true in task-specific dystonia), or was the only factor (as is thought to be possible in the yips). Due to the importance of the further study of dystonia in sports we decided to accept this limitation and include yips articles. Finally it is important to address the likely risk of publication bias towards positive results in the literature, as a further limitation to this review’s accuracy, and the likely strength of evidence for current treatments.

### 4.6 Conclusion

This systematic review showed a lack of evidence for any specific treatment of TSD in sports. The 31 studies reviewed, were of low quality and did not use standardized outcome measures and appropriate statistical analyses. A descriptive synthesis revealed emotional regulation was quite effective, but only applied to golfers. Almost no formal evidence to support the use of botulinum toxin or pharmacology was found. Future well designed studies in higher numbers of athletes and with sport specific outcome measures are essential to increase our understanding and improve treatment of TSD in sports.

## Author declaration

The authors affirm that the submission of this manuscript is in compliance with the following ethical requirements:

1. This study was conducted in strict accordance with the guidelines of Preferred Reporting Items for Systematic Reviews and Meta-Analyses (PRISMA), and was submitted to PROSPERO (Registration number: CRD42021261631).
2. Informed consent was not necessary for this work.
3. We confirm that we have read the position of Parkinsonism and Related Disorders on ethical publication and affirm that this work is consistent with those guidelines. The authors declare that the manuscript is original, it is not being considered for publication elsewhere and will not be submitted elsewhere while under consideration for Parkinsonism and Related Disorders or after it has been accepted by Parkinsonism & Related disorders. Authors declare there was also no ghost writing by anyone not named in the author list.
4. The authors have seen and approved the manuscript in the form it is being submitted to the journal. The authors declare that they have conformed to the highest standards of ethical conduct in the submission of accurate data and that they acknowledge the work of others when applicable.

## CRediT authorship contribution statement

**B. Nijenhuis:** Conceptualization, Data curation, Writing - original draft, Writing review & editing, Visualization, Investigation, Formal analysis, Methodology. **E. van Wensen:** Conceptualization, Data curation, Visualization, Writing - review & editing, Investigation, Methodology. **M. Smit:** Conceptualization, Methodology, Data curation. **T. van Zutphen:** Conceptualization, Funding acquisition, Writing - review & editing. **J. Zwerver:** Conceptualization, Funding acquisition, Data curation, Writing - review & editing, Validation Supervision, Project administration. **M.A.J. Tijssen:** Conceptualization, Funding acquisition, Data curation, Writing-review & editing, Validation, Supervision, Project administration.

## Declaration of competing interest

The authors declare that they have no known competing financial interests or personal relationships that could have appeared to influence the work reported in this paper.

## Figures and Tables

**Table:** 

**Item**	**Criteria**
1. Purpose	Was the purpose of the study clearly stated?
2. Literature review	Was relevant background literature reviewed to justify the intervention in task specific dystonia in sports for this study?
3. Study Design	What was the study design?
4. Blinding	Have the authors used assessor blinding to minimize bias?
5. Sample description	Have the authors described the sample in detail (i.e. age, gender, symptom duration, sport, diagnosis)?
6. Sample size	Was sample size justified (power calculation or post-hoc analysis)?
7. Ethics and consent	Have the authors documented ethical approval, and was informed consent obtained ?
8. Validity of outcomes	Did authors use ‘valid’ outcome measures, such that measures assessed the major elements of task specific dystonia clearly and specifically?
9. Reliability of outcomes	Did authors use ‘reliable’ outcome measures for interventions in task specific dystonia that had sufficient test-retest reliability or inter-rater reliability?
10. Intervention description	Did authors describe the intervention in sufficient detail to allow for replication?
11. Statistical significance	Was at least one result reported in terms of its statistical significance?
12. Statistical analysis	Were the statistical analyses employed appropriate to measure the efficacy of the interventions in task specific dystonia ?
13. Clinical importance	Did authors consider the clinical importance of the results for those with task specific dystonia?
14. Conclusions	Were the authors conclusions appropriate regarding the methods and results of the paper?
15. Clinical implications	Did authors consider the clinical implications of the interventions used in treating focal task specific dystonia in sports in directing future research?
16. Study limitations	Was there sufficient time spent outlining the limitations of the methodology and results of the study?

**Table:** 

**Study**	**Individual Item**																**/16**	**Descriptor**
	**1**	**2**	**3**	**4**	**5**	**6**	**7**	**8**	**9**	**10**	**11**	**12**	**13**	**14**	**15**	**16**		
Adler et al.	1	1	OL	0	1	0	1	1	1	1	1	1	1	1	1	1	**13**	**High**
Ahmad et al.	1	1	CS	0	1	0	1	1	1	1	0	0	1	1	0	1	**10**	**Moderate**
Bell et al 2007	1	1	CR	0	1	0	0	0	0	0	0	0	1	1	1	1	**7**	**Low**
Bell et al 2009	1	1	CS	0	1	0	1	0	0	1	0	0	1	1	1	1	**9**	**Moderate**
Bell et al 2011	1	1	CS	0	1	0	1	0	0	1	0	0	1	1	1	1	**9**	**Moderate**
Bennet et al.	1	1	SCS	0	1	0	1	1	1	1	0	0	1	1	1	1	**11**	**Moderate**
Kobori et al.	1	1	SCS	0	1	0	0	0	0	1	0	0	1	1	1	1	**8**	**Low**
Marquardt et al.	1	1	CR	0	0	0	0	1	1	0	0	0	1	1	1	0	**7**	**Low**
McClinton et al.	1	1	SCS	0	1	0	1	1	1	1	0	0	1	1	1	1	**11**	**Moderate**
Morrison et al.	1	1	SCS	0	1	0	0	1	1	1	1	1	1	1	0	1	**7**	**Low**
Rosted et al.	0	1	CR	0	1	0	0	0	0	1	0	0	1	1	1	1	**10**	**Moderate**
Rotherham et al.	1	1	SCS	0	0	0	0	0	0	0	0	0	1	1	1	1	**6**	**Low**
Suzuki et al.	1	1	CR	0	1	0	0	0	0	1	0	0	1	1	1	0	**7**	**Low**

**Table:** 

**Study**	**Individual Item**																**/16**	**Descriptor**
	**1**	**2**	**3**	**4**	**5**	**6**	**7**	**8**	**9**	**10**	**11**	**12**	**13**	**14**	**15**	**16**		
Adler et al.	1	1	OL	0	1	0	1	1	1	1	1	1	1	1	1	1	**13**	**High**
Ahmad et al.	1	1	CS	0	1	0	1	1	1	1	0	0	1	1	0	1	**10**	**Moderate**
Asahi et al.	1	1	SCS	0	1	0	1	0	0	1	0	0	1	1	0	0	**7**	**Low**
Barash et al.	0	0	CR	0	1	0	0	0	0	1	0	0	0	1	0	0	**3**	**Low**
Dhungana et al.	1	1	CS	0	1	0	1	0	0	1	0	0	1	1	1	0	**8**	**Low**
Garcia-Ruiz et al.	0	1	CR	0	1	0	0	0	0	0	0	0	0	1	0	0	**3**	**Low**
Horisawa et al.	1	1	SCS	0	1	0	0	0	0	1	0	0	1	1	1	0	**7**	**Low**
Horisawa et al.	1	1	MCS	0	0	0	1	1	1	1	0	1	1	1	1	0	**10**	**Moderate**
Jones et al.	0	0	CR	0	1	0	0	0	0	1	0	0	0	1	0	0	**3**	**Low**
Lee et al.	0	0	CR	0	1	0	1	0	0	1	0	0	0	1	0	0	**4**	**Low**
Leveille et al.	0	1	CS	0	1	0	0	0	0	1	0	0	0	1	1	0	**5**	**Low**
Mayer et al.	0	1	CR	0	1	0	0	0	0	1	0	0	1	0	0	0	**4**	**Low**
McClinton et al.	1	1	SCS	0	1	0	1	1	1	1	1	1	1	1	1	1	**13**	**High**
Nakane et al.	0	1	CS	0	1	0	0	0	0	1	0	0	1	1	1	0	**6**	**Low**
Ramdhani et al.	1	1	CS	0	1	0	0	0	1	0	0	0	1	1	1	0	**7**	**Low**
Ramirez-Gomez et al.	1	1	CR	0	0	0	0	0	0	0	0	0	0	1	0	0	**3**	**Low**
Ringman et al.	0	1	CR	0	1	0	0	0	0	1	0	0	1	0	0	0	**4**	**Low**
Sitburana et al.	1	1	CR	0	1	0	0	0	0	1	0	0	0	0	0	0	**4**	**Low**
Smilow et al.	1	1	CR	0	1	0	0	0	0	1	0	0	1	0	0	0	**5**	**Low**
Wagle et al.	0	1	CR	0	1	0	1	0	0	1	0	0	1	1	1	0	**7**	**Low**
Wu et al.	0	1	CS	0	1	0	1	0	0	1	0	0	1	1	1	0	**7**	**Low**

**Table:** 

**AuthorStudy designFollow-up period**	**Participants**	**Intervention**	**Outcome Measures**	**Result**	**Conclusion**
Adler et al. (2020)OL60 Minutes	• N= 14 • Age= 61.4 (mean) 8.0(STD) for the dystonic group), 55.9 (mean) 10.1 (STD) for the non-dystonic group • Symptom Duration= Not known • Sex= M • Sport= Golf • Diagnosis= Yips	1. Looking at the hole while putting	Standardized: **Y**Methods:* Surface EMG* SAM PuttlabFollow-up period:* 60 minutes.	1. Looking at the hole while putting: reduced symptoms (p < 0.001)	1. First to show quantified improvement2. Small sample size
Ahmad et al. (2018)CSNA	• N= 13 • Age at onset = 50.5 (mean) 8.8 (STD) • Symptom Duration= 5 yrs (mean) 1-25 yrs • Sex= 6 F, 7 M • Sport= Running • Diagnosis= Runner’s Dystonia	1. Physical therapy (1/13) 2. Weighted backpack (1/13)	Standardized: **N**Methods:* Subjective assessmentFollow-up period:* NA	1. Physical therapy: some improvement. 2. Weighted backpack: some improvement.	1. BoNT/A provided better relief2. Oral medication showed some results3. Small sample size4. No controls5. Retrospective
Bell et al.(2007)CR60 Days	• N= 1 • Age= 40 • Symptom Duration= 3 yrs • Sex= M • Sport= Golf • Diagnosis= Yips	4. Solution-focused guided imagery (SFGI)	Standardized: **Y**Methods:* Number of yips per round* Percentage of made putts within 4 feetFollow-up period:* 60 Days	4. Mean number of yips per round from 9.2 (before SFGI) to 0.2 (after SFGI)Percentage of made putts within 4 feet from 77% (before SFGI) to 81% (after SFGI)	1. May decrease the number of Yips and the number of putts per round of golf2. Case report
Bell et al.(2009)CS15 Weeks	• N= 3 • Age= 51 (mean) • Symptom Duration= not known • Sex= 3 M • Sport= Golf • Diagnosis= Yips	4. Solution focused guided imagery (SFGI)	Standardized: **Y**Methods:* Number of yips per round (9 holes)* Percentage of short putts with a yip (9 holes)Follow-up period:* 15 weeks	1. Mean number of yips per round from 3.5 (before SFGI) to 0.0 (after SFGI)2.Mean percentage of short putts with a yip in all 3 participants:35.5%, 30.6%, 36.7% (before SFGI) to 3 x 0.0% (after SFGI)	1. May decrease the number of Yips per round of golf2. Small sample size
Bell et al.(2011)CS12 Weeks	• N= 4 • Age= 51 (mean) • Symptom Duration= not known • Sex= 4 M • Sport= Golf • Diagnosis= Type I Yips	4. Solution focused guided imagery (SFGI)	Standardized: **Y**Methods:* N Number of yips per round (9 holes)* Video recording of all putting behaviorFollow-up period:* 12 weeks	1. Mean number of yips per round from 2.86 (before SFGI) to 0.08 (after SFGI)	1. May decrease the number of Yips per round of golf2. Small sample size
Bennett et al. (2017)SCS27 Weeks	• N= 1 • Age= 58 • Symptom Duration= 11 yrs • Sex= M • Sport= Golf • Diagnosis= Yips	4. Eye Movement and Desensitization Reprocessing (EMDR) in combination with exposure therapy (ET)	Standardized: **Y**Methods:* Number of 10 putts made from 3 and 4 feet* Visual inspection of figures of changing putter face rotation angle and putter face rotation angular velocity baseline and post interventionFollow-up period: 27 Weeks	1. Number of yips made from 3 feet 5/10 and from 4 feet 4/10 (before EMDR & ET) to 10/10 for both distances (after EMDR & ET))2. Visual inspection of the data from baseline to post intervention suggests in- creased control over the putting stroke following treatment	1. Previous life experiences might be associated with the onset of performance blocks and that EMDR with graded exposure might offer an effective treatment method2 Single case study
Kobori(2007)SCS8 Months	• N=1 • Age= 21 • Symptom Duration=1 • Sex= M • Sport= Rower • Diagnosis= Task Specific Focal Dystonia	1. Cognitive Behavioral Therapy (CBT): Explaining idiosyncratic model of anxiety and preoccupation to patient. Further behavioral and cognitive restructuring, psycho educating team members.	Standardized: NMethods:* Self-reported rating of anxiety and symptoms from 0-100Follow-up period:* 8 Months	2. Cognitive Behavioral Therapy (CBT): Self-reported ratings reduced from 95 to 30 and 90 to 15 for anxiety and symptom severity respectively between baseline and eight-month follow up.	1. Might be effective in treating TSD in rowing2. Single case study
Marquardt(2009)CRNA	• N= 1 • Age= NA • Symptom Duration=NA • Sex= NA • Sport= Golf • Diagnosis= Type 1 yips.	1. Sensory Motor Retraining: finding simpler unaffected related movement reintroducing original complexity step by step over 4 hours.	Standardized: **Y**Methods:* SAM Puttlab., not quantified* Subjective assessmentFollow-up period:* NA	1. Sensory motor retraining: SAM Puttlab showed rotation signals are now in a normal range. The issue almost disappeared. The wrist movements smooth and follow the putter movement.	1. Promising results in a yips-affected golfer2. Case report
McClinton et al.(2012)SCS1 Year	• N= 1 • Age=56 yrs • Symptom Duration:3yrs • Sex= M • Sport= Running • Diagnosis= Runner’s Dystonia	1. Sensory Motor Retraining 1: patient instructed to imagine moving his limb into dystonic positions 10x an hour. 2. Sensory Motor Retraining 2: Instructed to toe and heel-to-toe tap, attempting emulation of performance similar to uninvolved extremity.	Standardized: **Y**Methods:*Quantitative: 3-D kinematic/Dynamic EMG.*Survey: Lower Extremity Functional Scale (LEFS)*Subjective assessmentFollow-up period:* 1 Year	1. Sensory Motor Retraining: did not complete either protocol.	1. Some relief in a patient with a TSD in running2. Case report
Morrison & Milne (2015)SCS3 Months	• N= 1 • Age= 52 yrs • Symptom Duration= Unclear • Sex= Male • Sport= Golf • Diagnosis= Type 1 Yips	1. Pre-performance routine2. Positive self talk during shots/Breathing exercises.(Interventions took place over the course of approximately 2 months.)	Standardized: **Y**Methods:* Pre-performance routine (PPR) checklist* Bangor Sports Psychological Skills Inventory* Direct observation of chipping performance with statistical analysisFollow-up period:* 3 Months	1. Pre-performance routine: PPR checklist more complete (40 to 100 percent), 2. Bangor Sports Inventory improved and more balanced with higher self-reported confidence and motivation, 3. Chipping improved statistically significant with less frequent yips (from 65% to 15%)	1. May decrease the number of Yips and improve the golf chipping performance2. Psychological techniques that train golfers to concentrate more effectively appear to hold some promise in reducing yipping.3. Single case study
Rosted et al.(2005)CR24 Months	• N=1 • Age= 65 yrs • Symptom Duration: 2 yrs • Sex= M • Sport= Golf • Diagnosis= Yips	1. Acupuncture	Standardized: **N**Methods:* Follow up phone call with clinician at 6, 12 and 24 months post intervention.Follow-up period:* 24 Months	1. Acupuncture: patient reported symptoms disappearing after one session and sustained improvement over, 12 and 24 months	1. Acupuncture showed relief in a patient with a TSD in golf2. Case report
Rotheram et al. (2012)SCS6 Months	• N= 1 • Age= 49yrs • Symptom Duration=6 yrs • Sex= M • Sport= Golf • Diagnosis= Type I yips	1. Emotional Freedom Technique (EFT): 4 sessions two hours over 3 weeks.	Standardized: **Y**Methods:* Kinematics (SAM Putt Lab) and social validation questionnaire.* Performance: putting success rate.* Subjective assessment.Follow-up period: * 6 Months	1. EFT: Visual inspection of kinematics showed improvement. Putting success rate improved at six-month follow-up. Participants experienced emotional ‘freedom’ post intervention. Fewer visual occurrences of yips	1. Seemed effective in treating the TSD in golf: type I yips2. Single case study
Suzuki et al. (2011)CRNA	• N= 1 • Age= 59yrs • Symptom Duration= 6yrs • Sex= M • Sport= Running • Diagnosis= Task Specific Dystonia	1. Sensory trick: Holding hands over head. 2. Sensory trick:Running in a clockwise direction. 3. Sensory trick: imagining running in a clockwise direction.	Standardized: **N**Methods:*Video* Subjective assessmentFollow-up period:* NA	1. Holding hands over head: The symptoms disappeared. 2. Running clockwise direction: The orientation of his neck and trunk improved 3. Imagining running in a clockwise direction: symptoms improved.	1. Ameliorated trunk- & neck TSD in a runner2. Case report
**TherapyOL=Open, Label SCS= Single Case Study, MCS= Multiple Case Study, CR = Case Report, CS= Case Series NA= Not Applicable, NR=No Response.**					

**Table:** 

**AuthorStudy DesignFollow-up period**	**Participants**	**Intervention**	**Outcome Measures**	**Result**	**Conclusion**
Adler et al. (2020)OL60 Minutes	• N= 14 • Age= 61.4 (mean) 8.0(STD) for the dystonic group), 55.9 (mean) 10.1 (STD) for the non-dystonic group • Symptom Duration: Not known • Sex= M • Sport= Golf • Diagnosis= Yips	1. Propranolol 1 x 10 mg2. Looking at the hole while putting.	Standardized: **Y**Methods:* Surface EMG* Quantitative measurements of putter movement with a SAM PuttlabFollow-up period:* 60 minutes.	1. Propranolol: reduction in symptoms (p < 0.001)2. Looking at the hole while putting: reduced symptoms (p < 0.001)	1. First to show quantified improvement2. Small sample size
Ahmad et al. (2018)CSNA	• N= 13 • Age at onset = 50.5 (mean) 8.8 (STD) • Symptom Duration: 5 yrs (mean) 1-25 yrs (range) • Sex= 6 F, 7 M • Sport= Running • Diagnosis= Runner’s Dystonia	1. BTX -A (9/13) 2. Clonazepam (1/13), Levodopa (4/13), Carbamazepine (1/13) Trihexyphenidyl (1/13)	Standardized: **N**Methods:* Clinician’s assessmentFollow-up period:* NA	1. BTX-A: 2/9 NR, 4/9 some result, 3/9 significant result (70-90% improvement reported) 2. Clonazepam: some result Levodopa: NR, Carbamazepine: NR, Trihexyphenidyl: some result	1. BoNT/A provided better relief2. Oral medication showed some results3. Small sample size4. No controls5. Retrospective
Asahi et al. (2017)SCS6 Months	• N= 1 • Age= 20 • Symptom Duration: 1 yr • Skill Experience: 12 yrs • Sex= F • Sport= Table Tennis • Diagnosis= Task Specific Dystonia in left arm	1. Trihexyphenidyl: 12 mg daily 3 month, Clonazepam: 1 mg daily 3 month2. Ventro-oral thalamotomy right.	Standardized: **N**Methods:* Clinician’s assessmentFollow-up period:* 6 months.	1. Trihexyphenidyl: NRClonazepam: NR2. Ventro-oral thalamotomy right: complete remission.	1. Vo-thalamotomy seems effective in TSD in table tennis for at least 6 months2. Single Case Report
Barash et al. (2012)CRNA	• N= 1 • Age= 22 • Symptom Duration: < 1 yr • Sex= M • Sport= Rowing • Diagnosis= Rower's Dystonia	1. Levodopa	Standardized: **N**Methods:* Clinician’s assessmentFollow-up period:* NA	1. Levodopa: NR	1. Levo-dopa treatment for the TSD in rowing is not effective2. Case Report
Dhungana and Jankovic (2013)CSNA	• N= 2 • Age= 52, 62 • Symptom Duration: 30 yrs, not known • Sex= 2 M • Sport= Golf • Diagnosis= task-specific hand dystonia and task-specific cervical dystonia	1. BTX -A	Standardized: **N**Methods:* Clinician’s assessmentFollow-up period:* NA	1. BoNT/A was effective in treating the golfer’s yips	1. Good result in treating both participants 2. Small sample size
García-Ruiz et al. (2011)CRNA	• N= 1 • Age= 30 • Symptom Duration: 3 yrs • Sex= F • Sport= Flamenco dancing • Diagnosis= Dancers dystonia	1. Levodopa Benzodiazepines	Standardized: **N**Methods:* Clinician’s assessmentFollow-up period:* NA	1. Levodopa: NRBenzodiazepines: NR	1. Levodopa and Benzodiazepines had no effect in a Flamenco dancer’s dystonia2. Case Report
Horisawa et al. (2018)SCS18 Months	• N= 1 • Age= 38 • Symptom Duration: 3 yrs • Sex= M • Sport= Running, Bicycling • Diagnosis= Task Specific Lower Extremity Dystonia	1. Trihexyphenidyl 2. Ventro-oral thalamotomy: Two identical operations separated by 6 months (2nd operation had longer coagulation time 40 vs 30 seconds).	Standardized: **N**Methods:* Clinician’s assessment.Follow-up period:* 18 Months	1. Trihexyphenidyl: No result 2. Ventro-oral thalamotomy: Operation 1= First day post surgery symptoms disappeared while walking or bicycling. Two months after symptoms recurred. Operation 2= Symptoms disappeared, patient returned to running with no difficulties (Fig. 1C). After 3 months full recovery.	1. Vo-thalamotomy seems effective in the TSD in running and cycling for at least 18 months2. Single Case Study
Horisawa et al. (2019)MCSMean 47.36 months (range: 13-165)	• N= 4 • Age= 26 yrs (mean) • Symptom Duration= 5 yrs (mean) • Sex= M • Sport= 2 Tennis, 1 table tennis, 1 Juggling • Diagnosis=Task Specific Dystonia	1. Ventro-oral thalamotomy	Standardized: **Y**Methods:* The task specific focal dystonia scale (TFDS).Follow-up period:* NA	1. Ventro-oral thalamotomy TFDS scale improvement: Tennis player1: 2 to 5 Tennis player2: 2 to 5 (later recurrence 2-3)Table tennis player: 2 to 5 Juggler: 1 to 4.	1. Vo-thalamotomy seems to have long-term efficacy in the TSD in tennis, table tennis and juggling2. Vo-thalamotomyseems safe in TSD3. Retrospective study4. Small sample size
Jones et al. (2017)CRNA	• N= 1 • Age= 31 • Symptom Duration= Since his teens (not specific) • Sex= M • Sport= Baseball • Diagnosis=Focal limb dystonia	1. Trihexyphenidyl: 2 mg twice a day. Tetrabenazine: 25 mgdaily	Standardized: **N**Methods:* Clinician’s assessmentFollow-up period:* NA	1. Trihexyphenidyl NR Tetrabenazine: NR	1. No known treatment for the TSD in baseball 2. Case Report
Lee et al. (2021)CR18 Months	• N= 1 • Age= 52 yrs • Symptom Duration: 5 yrs • Skill Experience:30 yrs • Sex= M • Sport= Billiards • Diagnosis= Task Specific Dystonia	1. BTX -A: Right biceps, triceps, brachioradialis, and extensor carpi radialis muscles injected with 20, 20, 15, and 15 IU over 18 months at regular intervals 2. Propranolol BenzodiazepinesAnticholinergics	Standardized: **N**Methods:* Clinician’s assessmentFollow-up period:* 18 Months	1. BTX-A: Symptoms did not worsen. Patient not satisfied and refused further treatment. 2. Propranolol, a benzodiazepine and anticholinerges did not alleviate symptoms.	1. No known treatment for the TSD in billiards2. Case Report
Leveille et al. (2008)CS6 & 8 Months	• N= 2 • Age= 57 and 40 yrs • Symptom Duration: 2 and 10 yrs • Sex= F , M • Sport=Long distance and elite middle distance running • Diagnosis= Runner’s dystonia	1. BTX-A: Left tibialis posterior and intrinsic foot muscles. 100 units 4 times over 8 months (Case 1). Posterior tibialis muscle 50, 75, and 100 units over 6-months (Case 2). 2. Sinemet: Low dose (case 1). Standard Sinemet 25-100, one half tablet twice daily (case 2). 3. Bracing	Standardized: **N**Methods:* Clinician’s assessmentFollow-up period: case 1 = 8 months and case 2 = 6 months	1. BTX-A: Initial improvement in ambulation but still unable to run due to dystonia (case 1). Mild improvement following injections. 2. Sinemet: No benefit (case 1 and case 2). 3. Bracing: Combined with BTX-A, bracing was employed with some benefit. Allowing for running on rough terrain, but not on flat hard surfaces (case 2).	1. BoNT/A showed some relief in 2 patients with a TSD in running2. Sinemet showed no effect3. Small sample size
Mayer et al. (1999)CR3 Years	• N= 1 • Age= 34 yrs • Symptom Duration: 18 yrs • Skill Experience: 28 yrs • Sex= M • Sport= Tennis • Diagnosis= Segmental (Focal) Dystonia	1. Trihexyphenidyl-HCl: Initiated 12 mg·d−1. After improvement, therapy was first reduced to 5 mg. Artane retard, then to 2 × 5 mg Artane ret/day.	Standardized: **N**Methods:* Clinician’s assessmentFollow-up period:* 3 years.	1. Trihexyphenidyl-HCl: clear improvement in symptoms 3 yr after initiation of therapy. Improvement of athletic performance by 50-70% over baseline.	1.Trihexyphenidyl-HCl seems to have long-term efficacy in the TSD in tennis 2. Case report
McClinton et al. (2012)SCS1 Year	• N= 1 • Age=56 yrs • Symptom Duration: 3yrs • Sex= M • Sport= Running • Diagnosis= Runner’s Dystonia	1. BTX-A: Series of 3 injections 3 months apart in progressive doses (50, 100, and 150 U) to the left hamstring.2. Clonazepam	Standardized: **Y**:Methods:* Kinematics* EMG* Survey: Lower Extremity Functional Scale (LEFS)Follow-up period:* 1 year.	1. BTX-A: Improved score on LEFS (40/80 to 55/80). Patient reported no subjective change or improvement. No change in measures of EMG and Kinematics. 2. Clonazepam: Patient reported improvement in walking duration and less need of walking aids.	1 BoNT/A and oral medication showed some relief in a patient with a TSD in running2. Single case study
Nakane et al. (2018)CS1.5 Years and NA	• N=2 • Age= 15 and 41 yrs • Symptom Duration: 3 and 12 months • Skill Experience: 9 and 33 yrs • Sex= M • Sport= Baseball • Diagnosis= Yips	1. BTX-A: deltoid, trapezius, and supraspinatus muscles (case 2). 2. Lidocaine: deltoid, trapezius, and pectoralis major (0.5%). 5 times for 3 month (case 1).	Standardized: **N**Methods:* Clinician’s assessment (video analysis).Follow-up period:* case 1= 1.5 years* case 2= NA	1. BTX-A: produced weakness in the injected muscles. Unable to play baseball (case 2). 2. Lidocaine: significant improvement in his condition 1.5 yrs after onset. Dystonia persisted slightly (case 1).	1. Lidocaine injections seem to have long-term efficacy in the TSD in baseball2. Small sample size
Ramdhani and Frucht. (2013)CSNA	• 4 • Age= 36 yrs • Symptom Duration: 3.3 yrs • Sex= 4 F • Sport= Distance Walking/Running • Diagnosis= Task Specific Lower Limb Dystonia.	1. BoNT/A 2. Oral Medication: Sinemet, trihexyphenidyl-HCl, Baclofen, Valium, Klonopin.	Standardized: **N**Methods:* Clinician’s assessmentFollow-up period:* NA	1. BTX-A: unresponsive (1 case) 2. Oral Medication: a. Sinemet: NR (2 cases) b. Artane: NR (2 cases) c. Baclofen: NR (1 case) d. Valium: positive response (1 case) e. Klonopin: NR (1 case)	1. Valium had a positive respons in one patient with runner’s dystonia2. Small sample size
Ramìres Gómez et al. (2016)CR3 Weeks	• N= 1 • Age= 25 yrs • Symptom Duration: 2 yrs • Skill Experience: 5 yrs • Sex= M • Sport= Juggling • Diagnosis= Task Specific Dystonia	1. BTX-A: brachioradialis muscle, the superficial flexor (fingers) and the left flexor carpi muscle.	Standardized: **N**Methods:* Clinician’s assessmentFollow-up period:* 3 weeks	1. BTX-A: 60% improvement with complete alleviation for 15 minutes (no symptoms). Subject could continue as a juggler, albeit needing more frequent rest.	1. BoNT/A showed remarkable relief in a patient with a TSD in juggling2. Case report
Ringman et al. (2007)CRNA	• N= 1 • Age= 64 yrs • Symptom Duration: NA • Skill Experience: NA • Sex= M • Sport= Juggling • Diagnosis= Task Specific Dystonia	1. Memantine: 20 mg/day	Standardized: **N**Methods:* Clinician’s assessmentFollow-up period:* NA	2. Memantine: Substantial decrease in his yips symptoms.	1. Memantine showed substantial relief in a patient with a TSD in juggling2. Case report
Sitburana and Ondo. (2008)CR	• N= 1 • Age= 64 yrs • Sex= M • Sport= Pistol Shooting • Diagnosis= task specific pistol shooting dystonia	1. BTX-A: extensor carpi radialis (5units), pronator teres (25 units), flexor carpi radialis (40 units) and flexor carpi ulnaris (30 units).	Standardized: **N**Methods:* Clinician’s assessmentFollow-up period: * NA	1. NA	1. BoTN/A showed no relief in a patient with a TSD in pistol shooting2. Case report
Smilowska et al. (2019)CR9 Months	• N= 1 • 57 yrs • Symptom Duration: 5 yrs • Sex= M • Sport= Billiards • Diagnosis= Task Specific Dystonia	1. BTX-A: triceps brachii (20 units), biceps brachii (40 units) brachioradial (20 units), and flexor carpi radialis muscle (40 units).3 times over 3 months.	Standardized: **N**:Methods:* Clinician’s assessmentFollow-up period:* 9 Months	1. BTX-A: ‘Excellent’ improvement during the 3 month period after session 1 and 2 of BoNT/A injections. Last injection was less effective, but the patient was still able to play billiards.	1. BoTN/A showed remarkable relief in a patient with a TSD in billiards2. Case report
Wagle Shukla et al. (2018)CR18 Months	• N= 1 • Age= 56 yrs • Symptom Duration: 10 yrs. • Sex= M • Sport= Golf • Diagnosis= Task Specific Dystonia	1. Bilateral globus pallidus internus (GPi) deep brain stimulation(DBS) 2. BTX-A: left sternocleidomastoid, right splenius capitis, right semispinalis capitis, and bilateral trapezius (max dose 300 units). 3. Oral Medication: Baclofen, Tizanidine, clonazepam, and trihexyphenidyl.	Standardized: **N**Methods:* Clinician’s assessment (video analysis)Follow-up period:* 18 Months	1. Bilateral GPi DBS: 50% improvement in symptoms at 3 months, 85% improvement after 6 months and sustained improvement after 18 months. Video analysis showed great improvement. Patient reported a resumption of playing competitive Golf. 2. BTX-A: No Response. 3. Oral Medication: No Response	1. Bilateral GPi DBS showed remarkable relief in a patient with a TSD in golf2. BoTN/A and oral medication showed no relief.3. Case report
Wu and Jankovic. (2006)CSNA	• N= 5 • Age= 45 yrs (mean) • Symptom Duration: 7.2 ±4.4 yrs (mean) • Sex= 3F 2M • Sport= Distance Running • Diagnosis= Yips(type 1)	1. BTX-A: injection left iliopsoas (150 U), vastus medialis (150 U), and rectus femoris (150 U) 2. Oral Medication: Carbamazepine (400 mg/day), Levodopa, Trihexyphenidyl. 3. Hypnotherapy.	Standardized: **N**Methods:* Clinician’s assessment (video analysis)Follow-up period: * NA	1. BTX-A: Positive response. Resumed cycling competitions (3 yrs). 2. Oral Medication: Carbamazepine: improvement in 2 days and walks normally. Levodopa / Trihexyphenidyl improved symptoms. 3. Hypnotherapy: No response.	1. BoNT/A showed relief in patients with runner’s dystonia2. Oral medication improved symptoms3. Small sample size
**TherapyOL=Open, Label SCS= Single Case Study, MCS= Multiple Case Study, CR = Case Report, CS= Case Series NA= Not Applicable, NR=No Response.**					

## Appendix A

Search Protocol for Search Engines:¶

• PubMed-search:¶
  o (((“task-specific”[tiab] OR occupational[tiab] OR golf*[tiab] OR baseball[tiab] OR billiard[tiab] OR runner*[tiab] OR skat*[tiab] OR tennis*[tiab] OR petanque[tiab] OR sport*[tiab] OR “Sports”[Mesh] OR “athletic performance*”[tiab] OR “cardiorespiratory fitness”[tiab] OR “physical endurance*”[tiab] OR “physical fitness”[tiab] OR basketball[tiab] OR bicycling[tiab] OR boxing[tiab] OR cricket[tiab] OR football[tiab] OR gymnastic*[tiab] OR hockey[tiab] OR “martial art”[tiab] OR “tai ji”[tiab] OR mountaineering[tiab] OR racquet*[tiab] OR running[tiab] OR jogging[tiab] OR snow*[tiab] OR skiing[tiab] OR soccer[tiab] OR “track and field”[tiab] OR volleyball[tiab] OR walking[tiab] OR water[tiab] OR swimming[tiab] OR “weight lifting*”[tiab] OR wrestling[tiab] OR atlete*[tiab] OR judo[tiab] OR weightlifting*[tiab]) AND (dystoni*[tiab] OR cramp*[tiab])) OR pseudodystonia*[tiab] OR yips[tiab] OR “choking under pressure”[tiab] OR “Dystonia, Focal, Task-Specific” [Supplementary Concept])¶
• EMBASE-search:¶
  o ((('sport'/exp OR (task-specific OR occupational OR golf* OR baseball OR billiard OR runner* OR skat* OR tennis* OR petanque OR sport* OR ‘athletic performance*’ OR ‘cardiorespiratory fitness’ OR ‘physical endurance*’ OR ‘physical fitness’ OR basketball OR bicycling OR boxing OR cricket OR football OR gymnastic* OR hockey OR ‘martial art*’ OR ‘tai ji’ OR mountaineering OR racquet* OR running OR jogging OR snow* OR skiing OR soccer OR ‘track and field’ OR volleyball OR walking OR water OR swimming OR ‘weight lifting*’ OR wrestling OR altlete* OR judo OR weightlifting*):ti,ab) AND (dystoni*:ti,ab OR cramp*:ti,ab)) OR (pseudodystonia* OR yips OR ‘choking under pressure’):ti,ab OR 'task specific dystonia'/exp)¶
• Web Of Science-search:¶
  o (((task-specific OR occupational OR golf* OR baseball OR billiard OR runner* OR skat* OR tennis* OR petanque OR sport* OR “athletic performance*” OR “cardiorespiratory fitness” OR “physical endurance*” OR “physical fitness” OR basketball OR bicycling OR boxing OR cricket OR football OR gymnastic* OR hockey OR “martial art*” OR “tai ji” OR mountaineering OR racquet* OR running OR jogging OR snow* OR skiing OR soccer OR “track and field” OR volleyball OR walking OR water OR swimming OR “weight lifting*” OR wrestling OR athlete* OR judo OR weightlifting*) AND (dystoni* OR cramp*)) OR pseudodystonia* OR yips OR “choking under pressure”)¶
• PsychINFO-search:¶
• (((DE “Sports” OR DE “Athletes” OR DE “Baseball” OR DE “Basketball” OR DE “Extreme Sports” OR DE “Football” OR DE “Judo” OR DE “Martial Arts” OR DE “Professional Sports” OR DE “Soccer” OR DE “Swimming” OR DE “Tennis” OR DE “Weightlifting” OR (TI task-specific OR AB task-specific) OR (TI occupational OR AB occupational) OR (TI golf* OR AB golf*) OR (TI baseball OR AB baseball) OR (TI billiard OR AB billiard) OR (TI runner* OR AB runner*) OR (TI skat* OR AB skat*) OR (TI tennis* OR AB tennis*) OR (TI petanque OR AB petanque) OR (TI sport* OR AB sport*) OR (TI “athletic performance*” OR AB “athletic performance*”) OR (TI athlete* OR AB athlete*) OR (TI judo OR AB judo) OR (TI “cardiorespiratory fitness” OR AB “cardiorespiratory fitness”) OR (TI “physical endurance*” OR AB “physical endurance*”) OR (TI “physical fitness” OR AB “physical fitness”) OR (TI basketball OR AB basketball) OR (TI bicycling OR AB bicycling) OR (TI boxing OR AB boxing) OR (TI cricket OR AB cricket) OR (TI football OR AB football) OR (TI gymnastic* OR AB gymnastic*) OR (TI hockey OR AB hockey) OR (TI “martial art*” OR AB “martial art*”) OR (TI “tai ji” OR AB “tai ji”) OR (TI mountaineering OR AB mountaineering) OR (TI racquet* OR AB racquet*) OR (TI running OR AB running) OR (TI jogging OR AB jogging) OR (TI snow* OR AB snow*) OR (TI skiing OR AB skiing) OR (TI soccer OR AB soccer) OR (TI “track and field” OR AB “track and field”) OR (TI volleyball OR AB volleyball) OR (TI walking OR AB walking) OR (TI water OR AB water) OR (TI swimming OR AB swimming) OR (TI “weight lifting*” OR AB “weight lifting*”) OR (TI weightlifting* OR AB weightlifting*) OR (TI wrestling OR AB wrestling)) AND ((TI dystoni* OR AB dystoni*) OR (TI cramp* OR AB cramp*))) OR (TI pseudodystonia* OR AB pseudodystonia*) OR (TI yips OR AB yips) OR (TI “choking under pressure” OR AB “choking under pressure”))¶

## Appendix B

McMaster Critical Review Form for Quantitative Studies (MCRF) SurveyMcMaster Critical Review: Results of Studies involving interventions with Psychology, Sensory Motor Retraining and Physio-TherapyOL=Open, Label SCS= Single Case Study, MCS= Multiple Case Study, CR = Case Report, CS= Case Series NA= Not Applicable, NR= No Results

McMaster Critical Review: Results of Studies involving interventions with BTX-A, Pharmacology and Surgery

OL=Open, Label SCS= Single Case Study, MCS= Multiple Case Study, CR = Case Report, CS= Case Series NA= Not Applicable, NR= No Results

## Appendix C

Paramedical Interventions

## Appendix D

Medical Interventions
